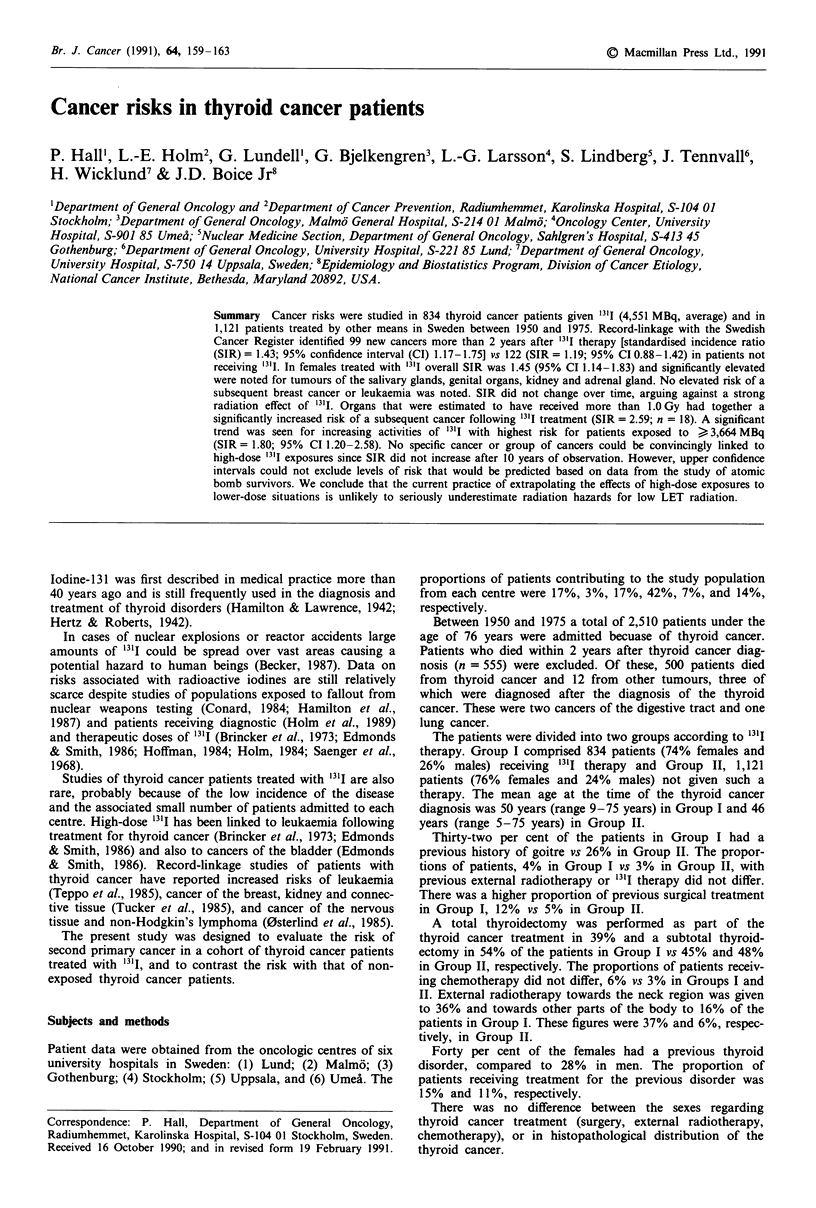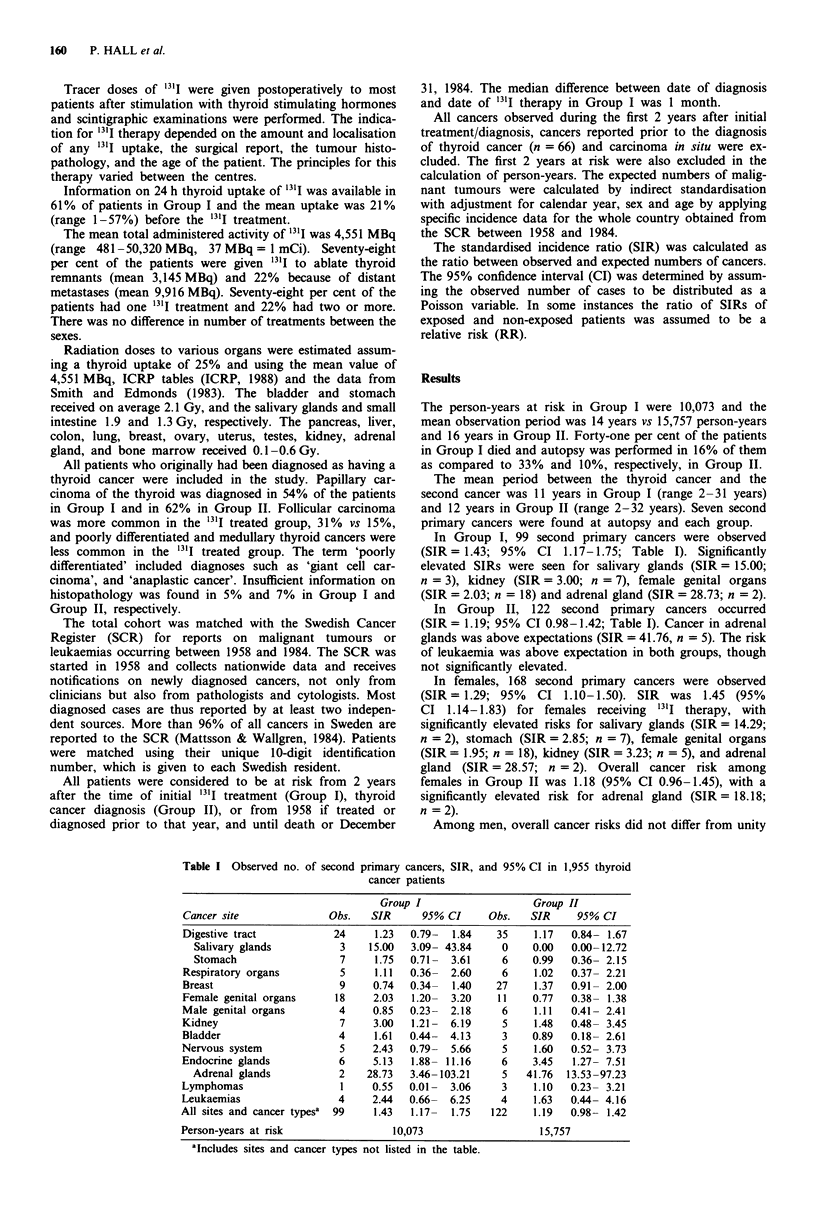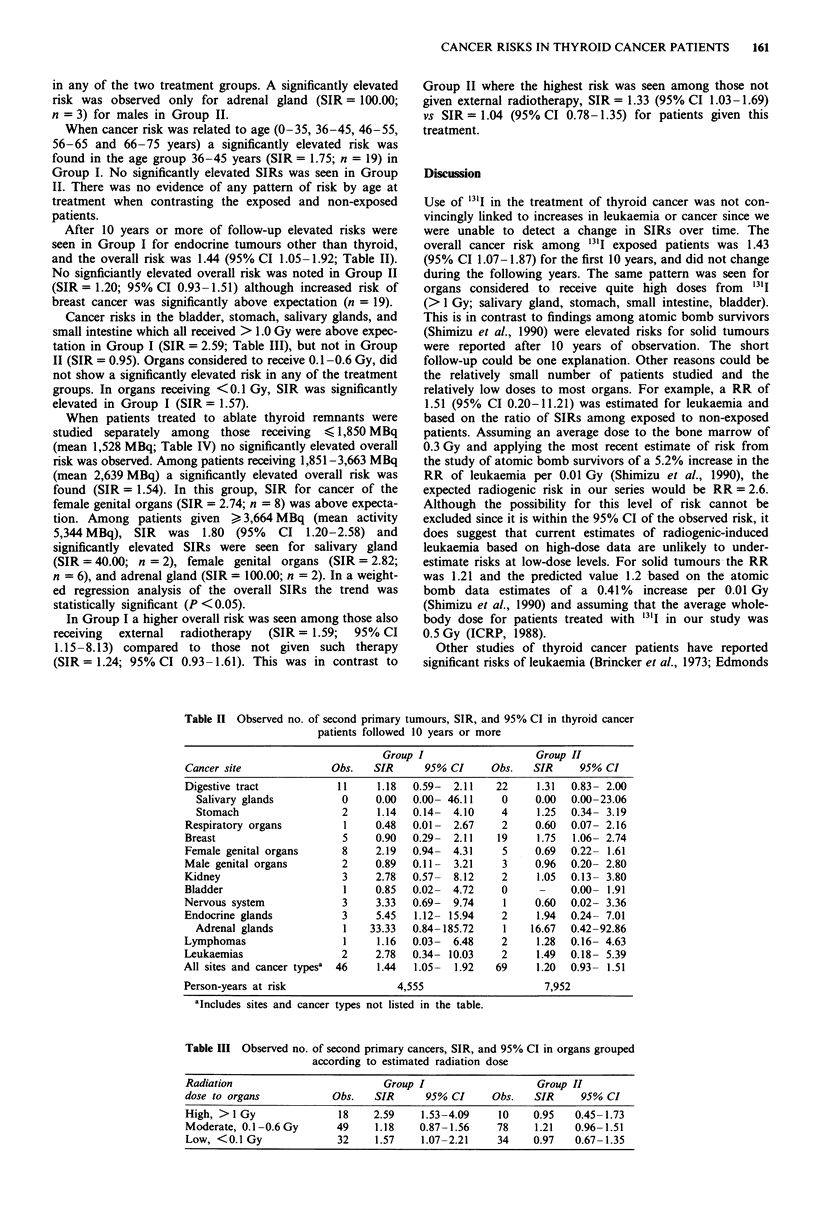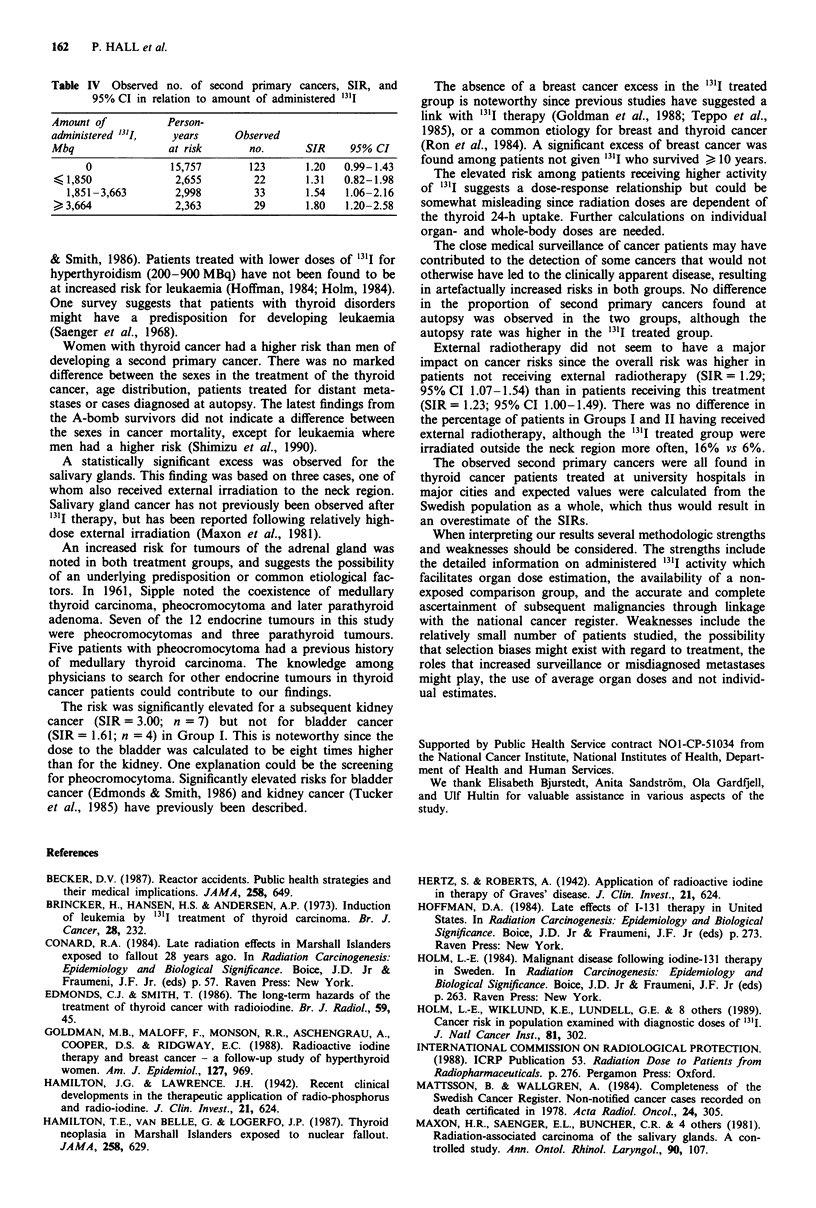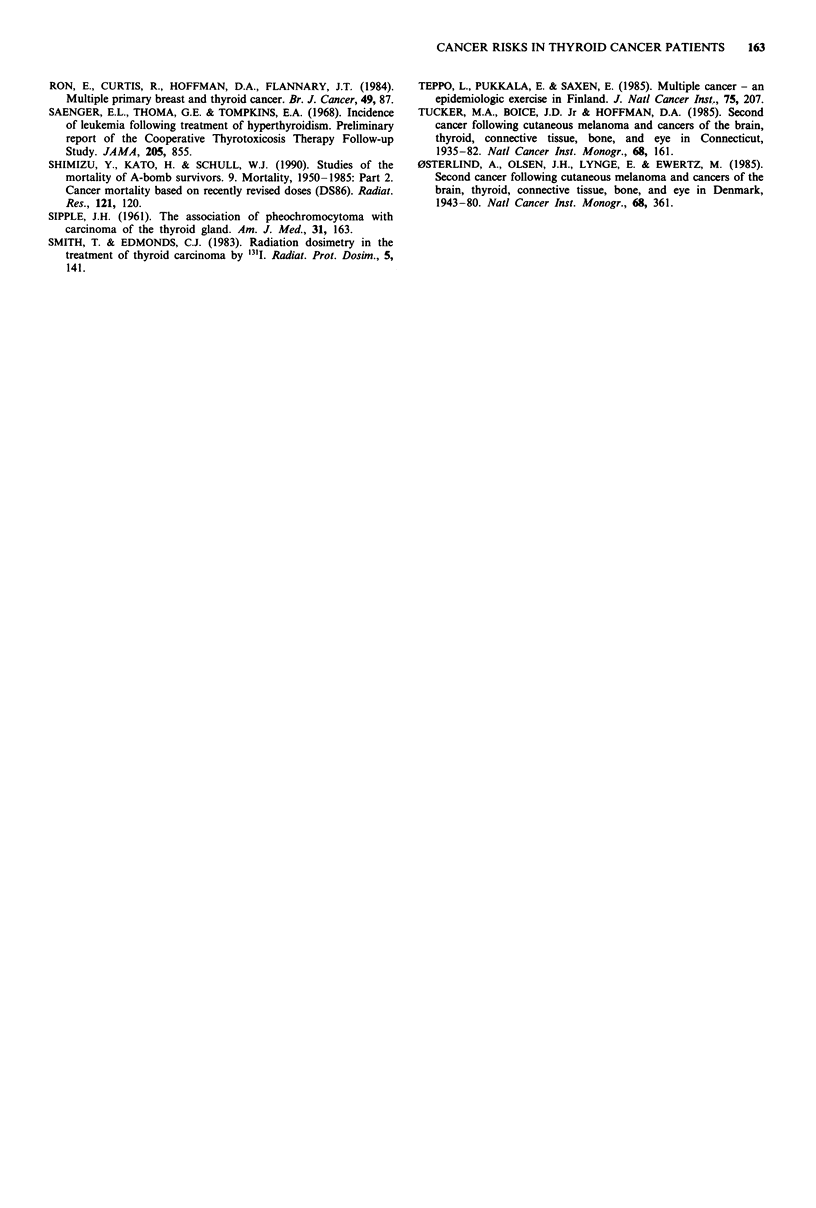# Cancer risks in thyroid cancer patients.

**DOI:** 10.1038/bjc.1991.261

**Published:** 1991-07

**Authors:** P. Hall, L. E. Holm, G. Lundell, G. Bjelkengren, L. G. Larsson, S. Lindberg, J. Tennvall, H. Wicklund, J. D. Boice

**Affiliations:** Department of General Oncology, Radiumhemmet, Karolinska Hospital, Stockholm, Sweden.

## Abstract

Cancer risks were studied in 834 thyroid cancer patients given 131I (4,551 MBq, average) and in 1,121 patients treated by other means in Sweden between 1950 and 1975. Record-linkage with the Swedish Cancer Register identified 99 new cancers more than 2 years after 131I therapy [standardised incidence ratio (SIR) = 1.43; 95% confidence interval (CI) 1.17-1.75] vs 122 (SIR = 1.19; 95% CI 0.88-1.42) in patients not receiving 131I. In females treated with 131I overall SIR was 1.45 (95% CI 1.14-1.83) and significantly elevated were noted for tumours of the salivary glands, genital organs, kidney and adrenal gland. No elevated risk of a subsequent breast cancer or leukaemia was noted. SIR did not change over time, arguing against a strong radiation effect of 131I. Organs that were estimated to have received more than 1.0 Gy had together a significantly increased risk of a subsequent cancer following 131I treatment (SIR = 2.59; n = 18). A significant trend was seen for increasing activities of 131I with highest risk for patients exposed to greater than or equal to 3,664 MBq (SIR = 1.80; 95% CI 1.20-2.58). No specific cancer or group of cancers could be convincingly linked to high-dose 131I exposures since SIR did not increase after 10 years of observation. However, upper confidence intervals could not exclude levels of risk that would be predicted based on data from the study of atomic bomb survivors. We conclude that the current practice of extrapolating the effects of high-dose exposures to lower-dose situations is unlikely to seriously underestimate radiation hazards for low LET radiation.


Br. J. Cancer (1991), 64, 159–163 © Macmillan Press Ltd., 1991

### Cancer risks in thyroid cancer patients

P. Hall[1], L.-E. Holm[2], G. Lundell[1], G. Bjelkengren[3], L.-G. Larsson[4], S. Lindberg[5], J. Tennvall[6], H. Wicklund[7] & J.D. Boice Jr[8]

[1]*Department of General Oncology and* [2]*Department of Cancer Prevention, Radiumhemmet, Karolinska Hospital, S-104 01 Stockholm;* [3]*Department of General Oncology, Malmö General Hospital, S-214 01 Malmö;* [4]*Oncology Center, University Hospital, S-901 85 Umeå;* [5]*Nuclear Medicine Section, Department of General Oncology, Sahlgren's Hospital, S-413 45 Gothenburg;* [6]*Department of General Oncology, University Hospital, S-221 85 Lund;* [7]*Department of General Oncology, University Hospital, S-750 14 Uppsala, Sweden;* [8]*Epidemiology and Biostatistics Program, Division of Cancer Etiology, National Cancer Institute, Bethesda, Maryland 20892, USA.*

**Summary** Cancer risks were studied in 834 thyroid cancer patients given $^{131}$I (4,551 MBq, average) and in 1,121 patients treated by other means in Sweden between 1950 and 1975. Record-linkage with the Swedish Cancer Register identified 99 new cancers more than 2 years after $^{131}$I therapy [standardised incidence ratio (SIR) = 1.43; 95% confidence interval (CI) 1.17–1.75] *vs* 122 (SIR = 1.19; 95% CI 0.88–1.42) in patients not receiving $^{131}$I. In females treated with $^{131}$I overall SIR was 1.45 (95% CI 1.14–1.83) and significantly elevated were noted for tumours of the salivary glands, genital organs, kidney and adrenal gland. No elevated risk of a subsequent breast cancer or leukaemia was noted. SIR did not change over time, arguing against a strong radiation effect of $^{131}$I. Organs that were estimated to have received more than 1.0 Gy had together a significantly increased risk of a subsequent cancer following $^{131}$I treatment (SIR = 2.59; $n$ = 18). A significant trend was seen for increasing activities of $^{131}$I with highest risk for patients exposed to $\geqslant$3,664 MBq (SIR = 1.80; 95% CI 1.20–2.58). No specific cancer or group of cancers could be convincingly linked to high-dose $^{131}$I exposures since SIR did not increase after 10 years of observation. However, upper confidence intervals could not exclude levels of risk that would be predicted based on data from the study of atomic bomb survivors. We conclude that the current practice of extrapolating the effects of high-dose exposures to lower-dose situations is unlikely to seriously underestimate radiation hazards for low LET radiation.

Iodine-131 was first described in medical practice more than 40 years ago and is still frequently used in the diagnosis and treatment of thyroid disorders (Hamilton & Lawrence, 1942; Hertz & Roberts, 1942).

In cases of nuclear explosions or reactor accidents large amounts of $^{131}$I could be spread over vast areas causing a potential hazard to human beings (Becker, 1987). Data on risks associated with radioactive iodines are still relatively scarce despite studies of populations exposed to fallout from nuclear weapons testing (Conard, 1984; Hamilton *et al.*, 1987) and patients receiving diagnostic (Holm *et al.*, 1989) and therapeutic doses of $^{131}$I (Brincker *et al.*, 1973; Edmonds & Smith, 1986; Hoffman, 1984; Holm, 1984; Saenger *et al.*, 1968).

Studies of thyroid cancer patients treated with $^{131}$I are also rare, probably because of the low incidence of the disease and the associated small number of patients admitted to each centre. High-dose $^{131}$I has been linked to leukaemia following treatment for thyroid cancer (Brincker *et al.*, 1973; Edmonds & Smith, 1986) and also to cancers of the bladder (Edmonds & Smith, 1986). Record-linkage studies of patients with thyroid cancer have reported increased risks of leukaemia (Teppo *et al.*, 1985), cancer of the breast, kidney and connective tissue (Tucker *et al.*, 1985), and cancer of the nervous tissue and non-Hodgkin's lymphoma (Østerlind *et al.*, 1985).

The present study was designed to evaluate the risk of second primary cancer in a cohort of thyroid cancer patients treated with $^{131}$I, and to contrast the risk with that of non-exposed thyroid cancer patients.

### Subjects and methods

Patient data were obtained from the oncologic centres of six university hospitals in Sweden: (1) Lund; (2) Malmö; (3) Gothenburg; (4) Stockholm; (5) Uppsala, and (6) Umeå. The proportions of patients contributing to the study population from each centre were 17%, 3%, 17%, 42%, 7%, and 14%, respectively.

Between 1950 and 1975 a total of 2,510 patients under the age of 76 years were admitted becuase of thyroid cancer. Patients who died within 2 years after thyroid cancer diagnosis ($n$ = 555) were excluded. Of these, 500 patients died from thyroid cancer and 12 from other tumours, three of which were diagnosed after the diagnosis of the thyroid cancer. These were two cancers of the digestive tract and one lung cancer.

The patients were divided into two groups according to $^{131}$I therapy. Group I comprised 834 patients (74% females and 26% males) receiving $^{131}$I therapy and Group II, 1,121 patients (76% females and 24% males) not given such a therapy. The mean age at the time of the thyroid cancer diagnosis was 50 years (range 9–75 years) in Group I and 46 years (range 5–75 years) in Group II.

Thirty-two per cent of the patients in Group I had a previous history of goitre *vs* 26% in Group II. The proportions of patients, 4% in Group I *vs* 3% in Group II, with previous external radiotherapy or $^{131}$I therapy did not differ. There was a higher proportion of previous surgical treatment in Group I, 12% *vs* 5% in Group II.

A total thyroidectomy was performed as part of the thyroid cancer treatment in 39% and a subtotal thyroidectomy in 54% of the patients in Group I *vs* 45% and 48% in Group II, respectively. The proportions of patients receiving chemotherapy did not differ, 6% *vs* 3% in Groups I and II. External radiotherapy towards the neck region was given to 36% and towards other parts of the body to 16% of the patients in Group I. These figures were 37% and 6%, respectively, in Group II.

Forty per cent of the females had a previous thyroid disorder, compared to 28% in men. The proportion of patients receiving treatment for the previous disorder was 15% and 11%, respectively.

There was no difference between the sexes regarding thyroid cancer treatment (surgery, external radiotherapy, chemotherapy), or in histopathological distribution of the thyroid cancer.

Correspondence: P. Hall, Department of General Oncology, Radiumhemmet, Karolinska Hospital, S-104 01 Stockholm, Sweden.
Received 16 October 1990; and in revised form 19 February 1991.

Tracer doses of $^{131}I$ were given postoperatively to most patients after stimulation with thyroid stimulating hormones and scintigraphic examinations were performed. The indication for $^{131}I$ therapy depended on the amount and localisation of any $^{131}I$ uptake, the surgical report, the tumour histopathology, and the age of the patient. The principles for this therapy varied between the centres.

Information on 24 h thyroid uptake of $^{131}I$ was available in 61% of patients in Group I and the mean uptake was 21% (range 1–57%) before the $^{131}I$ treatment.

The mean total administered activity of $^{131}I$ was 4,551 MBq (range 481–50,320 MBq, 37 MBq = 1 mCi). Seventy-eight per cent of the patients were given $^{131}I$ to ablate thyroid remnants (mean 3,145 MBq) and 22% because of distant metastases (mean 9,916 MBq). Seventy-eight per cent of the patients had one $^{131}I$ treatment and 22% had two or more. There was no difference in number of treatments between the sexes.

Radiation doses to various organs were estimated assuming a thyroid uptake of 25% and using the mean value of 4,551 MBq, ICRP tables (ICRP, 1988) and the data from Smith and Edmonds (1983). The bladder and stomach received on average 2.1 Gy, and the salivary glands and small intestine 1.9 and 1.3 Gy, respectively. The pancreas, liver, colon, lung, breast, ovary, uterus, testes, kidney, adrenal gland, and bone marrow received 0.1–0.6 Gy.

All patients who originally had been diagnosed as having a thyroid cancer were included in the study. Papillary carcinoma of the thyroid was diagnosed in 54% of the patients in Group I and in 62% in Group II. Follicular carcinoma was more common in the $^{131}I$ treated group, 31% *vs* 15%, and poorly differentiated and medullary thyroid cancers were less common in the $^{131}I$ treated group. The term 'poorly differentiated' included diagnoses such as 'giant cell carcinoma', and 'anaplastic cancer'. Insufficient information on histopathology was found in 5% and 7% in Group I and Group II, respectively.

The total cohort was matched with the Swedish Cancer Register (SCR) for reports on malignant tumours or leukaemias occurring between 1958 and 1984. The SCR was started in 1958 and collects nationwide data and receives notifications on newly diagnosed cancers, not only from clinicians but also from pathologists and cytologists. Most diagnosed cases are thus reported by at least two independent sources. More than 96% of all cancers in Sweden are reported to the SCR (Mattsson & Wallgren, 1984). Patients were matched using their unique 10-digit identification number, which is given to each Swedish resident.

All patients were considered to be at risk from 2 years after the time of initial $^{131}I$ treatment (Group I), thyroid cancer diagnosis (Group II), or from 1958 if treated or diagnosed prior to that year, and until death or December 31, 1984. The median difference between date of diagnosis and date of $^{131}I$ therapy in Group I was 1 month.

All cancers observed during the first 2 years after initial treatment/diagnosis, cancers reported prior to the diagnosis of thyroid cancer ($n = 66$) and carcinoma *in situ* were excluded. The first 2 years at risk were also excluded in the calculation of person-years. The expected numbers of malignant tumours were calculated by indirect standardisation with adjustment for calendar year, sex and age by applying specific incidence data for the whole country obtained from the SCR between 1958 and 1984.

The standardised incidence ratio (SIR) was calculated as the ratio between observed and expected numbers of cancers. The 95% confidence interval (CI) was determined by assuming the observed number of cases to be distributed as a Poisson variable. In some instances the ratio of SIRs of exposed and non-exposed patients was assumed to be a relative risk (RR).

### Results

The person-years at risk in Group I were 10,073 and the mean observation period was 14 years *vs* 15,757 person-years and 16 years in Group II. Forty-one per cent of the patients in Group I died and autopsy was performed in 16% of them as compared to 33% and 10%, respectively, in Group II.

The mean period between the thyroid cancer and the second cancer was 11 years in Group I (range 2–31 years) and 12 years in Group II (range 2–32 years). Seven second primary cancers were found at autopsy and each group.

In Group I, 99 second primary cancers were observed (SIR = 1.43; 95% CI 1.17–1.75; Table I). Significantly elevated SIRs were seen for salivary glands (SIR = 15.00; $n = 3$), kidney (SIR = 3.00; $n = 7$), female genital organs (SIR = 2.03; $n = 18$) and adrenal gland (SIR = 28.73; $n = 2$).

In Group II, 122 second primary cancers occurred (SIR = 1.19; 95% CI 0.98–1.42; Table I). Cancer in adrenal glands was above expectations (SIR = 41.76, $n = 5$). The risk of leukaemia was above expectation in both groups, though not significantly elevated.

In females, 168 second primary cancers were observed (SIR = 1.29; 95% CI 1.10–1.50). SIR was 1.45 (95% CI 1.14–1.83) for females receiving $^{131}I$ therapy, with significantly elevated risks for salivary glands (SIR = 14.29; $n = 2$), stomach (SIR = 2.85; $n = 7$), female genital organs (SIR = 1.95; $n = 18$), kidney (SIR = 3.23; $n = 5$), and adrenal gland (SIR = 28.57; $n = 2$). Overall cancer risk among females in Group II was 1.18 (95% CI 0.96–1.45), with a significantly elevated risk for adrenal gland (SIR = 18.18; $n = 2$).

Among men, overall cancer risks did not differ from unity

**Table I** Observed no. of second primary cancers, SIR, and 95% CI in 1,955 thyroid cancer patients

| | *Group I* | | | *Group II* | | |
|---|---|---|---|---|---|---|
| *Cancer site* | *Obs.* | *SIR* | *95% CI* | *Obs.* | *SIR* | *95% CI* |
| Digestive tract | 24 | 1.23 | 0.79– 1.84 | 35 | 1.17 | 0.84– 1.67 |
| Salivary glands | 3 | 15.00 | 3.09– 43.84 | 0 | 0.00 | 0.00–12.72 |
| Stomach | 7 | 1.75 | 0.71– 3.61 | 6 | 0.99 | 0.36– 2.15 |
| Respiratory organs | 5 | 1.11 | 0.36– 2.60 | 6 | 1.02 | 0.37– 2.21 |
| Breast | 9 | 0.74 | 0.34– 1.40 | 27 | 1.37 | 0.91– 2.00 |
| Female genital organs | 18 | 2.03 | 1.20– 3.20 | 11 | 0.77 | 0.38– 1.38 |
| Male genital organs | 4 | 0.85 | 0.23– 2.18 | 6 | 1.11 | 0.41– 2.41 |
| Kidney | 7 | 3.00 | 1.21– 6.19 | 5 | 1.48 | 0.48– 3.45 |
| Bladder | 4 | 1.61 | 0.44– 4.13 | 3 | 0.89 | 0.18– 2.61 |
| Nervous system | 5 | 2.43 | 0.79– 5.66 | 5 | 1.60 | 0.52– 3.73 |
| Endocrine glands | 6 | 5.13 | 1.88– 11.16 | 6 | 3.45 | 1.27– 7.51 |
| Adrenal glands | 2 | 28.73 | 3.46–103.21 | 5 | 41.76 | 13.53–97.23 |
| Lymphomas | 1 | 0.55 | 0.01– 3.06 | 3 | 1.10 | 0.23– 3.21 |
| Leukaemias | 4 | 2.44 | 0.66– 6.25 | 4 | 1.63 | 0.44– 4.16 |
| All sites and cancer types[a] | 99 | 1.43 | 1.17– 1.75 | 122 | 1.19 | 0.98– 1.42 |
| Person-years at risk | | 10,073 | | | 15,757 | |

[a]Includes sites and cancer types not listed in the table.

in any of the two treatment groups. A significantly elevated risk was observed only for adrenal gland (SIR = 100.00; $n = 3$) for males in Group II.

When cancer risk was related to age (0–35, 36–45, 46–55, 56–65 and 66–75 years) a significantly elevated risk was found in the age group 36–45 years (SIR = 1.75; $n = 19$) in Group I. No significantly elevated SIRs was seen in Group II. There was no evidence of any pattern of risk by age at treatment when contrasting the exposed and non-exposed patients.

After 10 years or more of follow-up elevated risks were seen in Group I for endocrine tumours other than thyroid, and the overall risk was 1.44 (95% CI 1.05–1.92; Table II). No signficiantly elevated overall risk was noted in Group II (SIR = 1.20; 95% CI 0.93–1.51) although increased risk of breast cancer was significantly above expectation ($n = 19$).

Cancer risks in the bladder, stomach, salivary glands, and small intestine which all received > 1.0 Gy were above expectation in Group I (SIR = 2.59; Table III), but not in Group II (SIR = 0.95). Organs considered to receive 0.1–0.6 Gy, did not show a significantly elevated risk in any of the treatment groups. In organs receiving <0.1 Gy, SIR was significantly elevated in Group I (SIR = 1.57).

When patients treated to ablate thyroid remnants were studied separately among those receiving ⩽1,850 MBq (mean 1,528 MBq; Table IV) no significantly elevated overall risk was observed. Among patients receiving 1,851–3,663 MBq (mean 2,639 MBq) a significantly elevated overall risk was found (SIR = 1.54). In this group, SIR for cancer of the female genital organs (SIR = 2.74; $n = 8$) was above expectation. Among patients given ⩾3,664 MBq (mean activity 5,344 MBq), SIR was 1.80 (95% CI 1.20–2.58) and significantly elevated SIRs were seen for salivary gland (SIR = 40.00; $n = 2$), female genital organs (SIR = 2.82; $n = 6$), and adrenal gland (SIR = 100.00; $n = 2$). In a weighted regression analysis of the overall SIRs the trend was statistically significant ($P < 0.05$).

In Group I a higher overall risk was seen among those also receiving external radiotherapy (SIR = 1.59; 95% CI 1.15–8.13) compared to those not given such therapy (SIR = 1.24; 95% CI 0.93–1.61). This was in contrast to Group II where the highest risk was seen among those not given external radiotherapy, SIR = 1.33 (95% CI 1.03–1.69) *vs* SIR = 1.04 (95% CI 0.78–1.35) for patients given this treatment.

### Discussion

Use of $^{131}$I in the treatment of thyroid cancer was not convincingly linked to increases in leukaemia or cancer since we were unable to detect a change in SIRs over time. The overall cancer risk among $^{131}$I exposed patients was 1.43 (95% CI 1.07–1.87) for the first 10 years, and did not change during the following years. The same pattern was seen for organs considered to receive quite high doses from $^{131}$I (> 1 Gy; salivary gland, stomach, small intestine, bladder). This is in contrast to findings among atomic bomb survivors (Shimizu *et al.*, 1990) were elevated risks for solid tumours were reported after 10 years of observation. The short follow-up could be one explanation. Other reasons could be the relatively small number of patients studied and the relatively low doses to most organs. For example, a RR of 1.51 (95% CI 0.20–11.21) was estimated for leukaemia and based on the ratio of SIRs among exposed to non-exposed patients. Assuming an average dose to the bone marrow of 0.3 Gy and applying the most recent estimate of risk from the study of atomic bomb survivors of a 5.2% increase in the RR of leukaemia per 0.01 Gy (Shimizu *et al.*, 1990), the expected radiogenic risk in our series would be RR = 2.6. Although the possibility for this level of risk cannot be excluded since it is within the 95% CI of the observed risk, it does suggest that current estimates of radiogenic-induced leukaemia based on high-dose data are unlikely to underestimate risks at low-dose levels. For solid tumours the RR was 1.21 and the predicted value 1.2 based on the atomic bomb data estimates of a 0.41% increase per 0.01 Gy (Shimizu *et al.*, 1990) and assuming that the average whole-body dose for patients treated with $^{131}$I in our study was 0.5 Gy (ICRP, 1988).

Other studies of thyroid cancer patients have reported significant risks of leukaemia (Brincker *et al.*, 1973; Edmonds

**Table II** Observed no. of second primary tumours, SIR, and 95% CI in thyroid cancer patients followed 10 years or more

| Cancer site | Group I Obs. | Group I SIR | Group I 95% CI | Group II Obs. | Group II SIR | Group II 95% CI |
|---|---|---|---|---|---|---|
| Digestive tract | 11 | 1.18 | 0.59– 2.11 | 22 | 1.31 | 0.83– 2.00 |
| Salivary glands | 0 | 0.00 | 0.00– 46.11 | 0 | 0.00 | 0.00–23.06 |
| Stomach | 2 | 1.14 | 0.14– 4.10 | 4 | 1.25 | 0.34– 3.19 |
| Respiratory organs | 1 | 0.48 | 0.01– 2.67 | 2 | 0.60 | 0.07– 2.16 |
| Breast | 5 | 0.90 | 0.29– 2.11 | 19 | 1.75 | 1.06– 2.74 |
| Female genital organs | 8 | 2.19 | 0.94– 4.31 | 5 | 0.69 | 0.22– 1.61 |
| Male genital organs | 2 | 0.89 | 0.11– 3.21 | 3 | 0.96 | 0.20– 2.80 |
| Kidney | 3 | 2.78 | 0.57– 8.12 | 2 | 1.05 | 0.13– 3.80 |
| Bladder | 1 | 0.85 | 0.02– 4.72 | 0 | – | 0.00– 1.91 |
| Nervous system | 3 | 3.33 | 0.69– 9.74 | 1 | 0.60 | 0.02– 3.36 |
| Endocrine glands | 3 | 5.45 | 1.12– 15.94 | 2 | 1.94 | 0.24– 7.01 |
| Adrenal glands | 1 | 33.33 | 0.84–185.72 | 1 | 16.67 | 0.42–92.86 |
| Lymphomas | 1 | 1.16 | 0.03– 6.48 | 2 | 1.28 | 0.16– 4.63 |
| Leukaemias | 2 | 2.78 | 0.34– 10.03 | 2 | 1.49 | 0.18– 5.39 |
| All sites and cancer types[a] | 46 | 1.44 | 1.05– 1.92 | 69 | 1.20 | 0.93– 1.51 |
| Person-years at risk | | 4,555 | | | 7,952 | |

[a]Includes sites and cancer types not listed in the table.

**Table III** Observed no. of second primary cancers, SIR, and 95% CI in organs grouped according to estimated radiation dose

| Radiation dose to organs | Group I Obs. | Group I SIR | Group I 95% CI | Group II Obs. | Group II SIR | Group II 95% CI |
|---|---|---|---|---|---|---|
| High, >1 Gy | 18 | 2.59 | 1.53–4.09 | 10 | 0.95 | 0.45–1.73 |
| Moderate, 0.1–0.6 Gy | 49 | 1.18 | 0.87–1.56 | 78 | 1.21 | 0.96–1.51 |
| Low, <0.1 Gy | 32 | 1.57 | 1.07–2.21 | 34 | 0.97 | 0.67–1.35 |

**Table IV** Observed no. of second primary cancers, SIR, and 95% CI in relation to amount of administered $^{131}$I

| *Amount of administered $^{131}I$, Mbq* | *Person-years at risk* | *Observed no.* | *SIR* | *95% CI* |
|---|---|---|---|---|
| 0 | 15,757 | 123 | 1.20 | 0.99–1.43 |
| ⩽1,850 | 2,655 | 22 | 1.31 | 0.82–1.98 |
| 1,851–3,663 | 2,998 | 33 | 1.54 | 1.06–2.16 |
| ⩾3,664 | 2,363 | 29 | 1.80 | 1.20–2.58 |

& Smith, 1986). Patients treated with lower doses of $^{131}$I for hyperthyroidism (200–900 MBq) have not been found to be at increased risk for leukaemia (Hoffman, 1984; Holm, 1984). One survey suggests that patients with thyroid disorders might have a predisposition for developing leukaemia (Saenger *et al.*, 1968).

Women with thyroid cancer had a higher risk than men of developing a second primary cancer. There was no marked difference between the sexes in the treatment of the thyroid cancer, age distribution, patients treated for distant metastases or cases diagnosed at autopsy. The latest findings from the A-bomb survivors did not indicate a difference between the sexes in cancer mortality, except for leukaemia where men had a higher risk (Shimizu *et al.*, 1990).

A statistically significant excess was observed for the salivary glands. This finding was based on three cases, one of whom also received external irradiation to the neck region. Salivary gland cancer has not previously been observed after $^{131}$I therapy, but has been reported following relatively high-dose external irradiation (Maxon *et al.*, 1981).

An increased risk for tumours of the adrenal gland was noted in both treatment groups, and suggests the possibility of an underlying predisposition or common etiological factors. In 1961, Sipple noted the coexistence of medullary thyroid carcinoma, pheocromocytoma and later parathyroid adenoma. Seven of the 12 endocrine tumours in this study were pheocromocytomas and three parathyroid tumours. Five patients with pheocromocytoma had a previous history of medullary thyroid carcinoma. The knowledge among physicians to search for other endocrine tumours in thyroid cancer patients could contribute to our findings.

The risk was significantly elevated for a subsequent kidney cancer (SIR = 3.00; $n = 7$) but not for bladder cancer (SIR = 1.61; $n = 4$) in Group I. This is noteworthy since the dose to the bladder was calculated to be eight times higher than for the kidney. One explanation could be the screening for pheocromocytoma. Significantly elevated risks for bladder cancer (Edmonds & Smith, 1986) and kidney cancer (Tucker *et al.*, 1985) have previously been described.

The absence of a breast cancer excess in the $^{131}$I treated group is noteworthy since previous studies have suggested a link with $^{131}$I therapy (Goldman *et al.*, 1988; Teppo *et al.*, 1985), or a common etiology for breast and thyroid cancer (Ron *et al.*, 1984). A significant excess of breast cancer was found among patients not given $^{131}$I who survived ⩾10 years.

The elevated risk among patients receiving higher activity of $^{131}$I suggests a dose-response relationship but could be somewhat misleading since radiation doses are dependent of the thyroid 24-h uptake. Further calculations on individual organ- and whole-body doses are needed.

The close medical surveillance of cancer patients may have contributed to the detection of some cancers that would not otherwise have led to the clinically apparent disease, resulting in artefactually increased risks in both groups. No difference in the proportion of second primary cancers found at autopsy was observed in the two groups, although the autopsy rate was higher in the $^{131}$I treated group.

External radiotherapy did not seem to have a major impact on cancer risks since the overall risk was higher in patients not receiving external radiotherapy (SIR = 1.29; 95% CI 1.07–1.54) than in patients receiving this treatment (SIR = 1.23; 95% CI 1.00–1.49). There was no difference in the percentage of patients in Groups I and II having received external radiotherapy, although the $^{131}$I treated group were irradiated outside the neck region more often, 16% *vs* 6%.

The observed second primary cancers were all found in thyroid cancer patients treated at university hospitals in major cities and expected values were calculated from the Swedish population as a whole, which thus would result in an overestimate of the SIRs.

When interpreting our results several methodologic strengths and weaknesses should be considered. The strengths include the detailed information on administered $^{131}$I activity which facilitates organ dose estimation, the availability of a non-exposed comparison group, and the accurate and complete ascertainment of subsequent malignancies through linkage with the national cancer register. Weaknesses include the relatively small number of patients studied, the possibility that selection biases might exist with regard to treatment, the roles that increased surveillance or misdiagnosed metastases might play, the use of average organ doses and not individual estimates.

Supported by Public Health Service contract NO1-CP-51034 from the National Cancer Institute, National Institutes of Health, Department of Health and Human Services.

We thank Elisabeth Bjurstedt, Anita Sandström, Ola Gardfjell, and Ulf Hultin for valuable assistance in various aspects of the study.

### References

BECKER, D.V. (1987). Reactor accidents. Public health strategies and their medical implications. *JAMA*, **258**, 649.

BRINCKER, H., HANSEN, H.S. & ANDERSEN, A.P. (1973). Induction of leukemia by $^{131}$I treatment of thyroid carcinoma. *Br. J. Cancer*, **28**, 232.

CONARD, R.A. (1984). Late radiation effects in Marshall Islanders exposed to fallout 28 years ago. In *Radiation Carcinogenesis: Epidemiology and Biological Significance*. Boice, J.D. Jr & Fraumeni, J.F. Jr. (eds) p. 57. Raven Press: New York.

EDMONDS, C.J. & SMITH, T. (1986). The long-term hazards of the treatment of thyroid cancer with radioiodine. *Br. J. Radiol.*, **59**, 45.

GOLDMAN, M.B., MALOFF, F., MONSON, R.R., ASCHENGRAU, A., COOPER, D.S. & RIDGWAY, E.C. (1988). Radioactive iodine therapy and breast cancer – a follow-up study of hyperthyroid women. *Am. J. Epidemiol.*, **127**, 969.

HAMILTON, J.G. & LAWRENCE. J.H. (1942). Recent clinical developments in the therapeutic application of radio-phosphorus and radio-iodine. *J. Clin. Invest.*, **21**, 624.

HAMILTON, T.E., VAN BELLE, G. & LOGERFO, J.P. (1987). Thyroid neoplasia in Marshall Islanders exposed to nuclear fallout. *JAMA*, **258**, 629.

HERTZ, S. & ROBERTS, A. (1942). Application of radioactive iodine in therapy of Graves' disease. *J. Clin. Invest.*, **21**, 624.

HOFFMAN, D.A. (1984). Late effects of I-131 therapy in United States. In *Radiation Carcinogenesis: Epidemiology and Biological Significance*. Boice, J.D. Jr & Fraumeni, J.F. Jr (eds) p. 273. Raven Press: New York.

HOLM, L.-E. (1984). Malignant disease following iodine-131 therapy in Sweden. In *Radiation Carcinogenesis: Epidemiology and Biological Significance*. Boice, J.D. Jr & Fraumeni, J.F. Jr (eds) p. 263. Raven Press: New York.

HOLM, L.-E., WIKLUND, K.E., LUNDELL, G.E. & 8 others (1989). Cancer risk in population examined with diagnostic doses of $^{131}$I. *J. Natl Cancer Inst.*, **81**, 302.

INTERNATIONAL COMMISSION ON RADIOLOGICAL PROTECTION. (1988). ICRP Publication 53. *Radiation Dose to Patients from Radiopharmaceuticals*. p. 276. Pergamon Press: Oxford.

MATTSSON, B. & WALLGREN, A. (1984). Completeness of the Swedish Cancer Register. Non-notified cancer cases recorded on death certificated in 1978. *Acta Radiol. Oncol.*, **24**, 305.

MAXON, H.R., SAENGER, E.L., BUNCHER, C.R. & 4 others (1981). Radiation-associated carcinoma of the salivary glands. A controlled study. *Ann. Ontol. Rhinol. Laryngol.*, **90**, 107.

RON, E., CURTIS, R., HOFFMAN, D.A., FLANNARY, J.T. (1984). Multiple primary breast and thyroid cancer. *Br. J. Cancer*, **49**, 87.

SAENGER, E.L., THOMA, G.E. & TOMPKINS, E.A. (1968). Incidence of leukemia following treatment of hyperthyroidism. Preliminary report of the Cooperative Thyrotoxicosis Therapy Follow-up Study. *JAMA*, **205**, 855.

SHIMIZU, Y., KATO, H. & SCHULL, W.J. (1990). Studies of the mortality of A-bomb survivors. 9. Mortality, 1950–1985: Part 2. Cancer mortality based on recently revised doses (DS86). *Radiat. Res.*, **121**, 120.

SIPPLE, J.H. (1961). The association of pheochromocytoma with carcinoma of the thyroid gland. *Am. J. Med.*, **31**, 163.

SMITH, T. & EDMONDS, C.J. (1983). Radiation dosimetry in the treatment of thyroid carcinoma by $^{131}$I. *Radiat. Prot. Dosim.*, **5**, 141.

TEPPO, L., PUKKALA, E. & SAXEN, E. (1985). Multiple cancer – an epidemiologic exercise in Finland. *J. Natl Cancer Inst.*, **75**, 207.

TUCKER, M.A., BOICE, J.D. Jr & HOFFMAN, D.A. (1985). Second cancer following cutaneous melanoma and cancers of the brain, thyroid, connective tissue, bone, and eye in Connecticut, 1935–82. *Natl Cancer Inst. Monogr.*, **68**, 161.

ØSTERLIND, A., OLSEN, J.H., LYNGE, E. & EWERTZ, M. (1985). Second cancer following cutaneous melanoma and cancers of the brain, thyroid, connective tissue, bone, and eye in Denmark, 1943–80. *Natl Cancer Inst. Monogr.*, **68**, 361.